# Health-related quality of life in pregnancy with uterine fibroid: a cross-sectional study in China

**DOI:** 10.1186/s12955-019-1153-6

**Published:** 2019-05-24

**Authors:** Wai-Kit Ming, Huailiang Wu, Yanxin Wu, Hanqing Chen, Tian Meng, Yiwei Shen, Ziyu Wang, Xinyu Huang, Weiwei Sun, Tik Sang Chow, Yuan Wang, Wenjing Ding, Haitian Chen, Zhuyu Li, Zilian Wang

**Affiliations:** 1 Department of Obstetrics and Gynaecology, The First Affiliated Hospital of Sun Yat-sen University, Guangzhou, China; 2 Pharmacoepidemiology and Pharmacoeconomic, Department of Medicine, Brigham and Women’s Hospital and Harvard Medical School, Boston, MA USA; 3 School of Medicine, Jinan University, Guangzhou, China

## Abstract

**Background:**

Uterine fibroids (UFs) are the most common benign tumors in women. They are likely to cause numerous clinical symptoms, such as pain, menorrhagia, and other obstetric complications in pregnant women. This study aimed to determine the health-related quality of life (HRQoL) during pregnancy with uterine fibroids (UF), thus providing a utility-based case value in pregnant women with UF and understanding of whether HRQoL is associated with clinical outcomes in pregnant women with UFs.

**Method:**

This study was conducted in a cross-sectional manner. This study was based on questionnaire surveys completed by sequential out- and in-patients and was conducted in a regional university hospital in Guangzhou, China. The EuroQoL five-dimension-five-level (EQ-5D-5 L) questionnaire was used, and demographic data were collected. An electronic record of the clinical outcomes of pregnant women with UF was retrieved from the hospital’s electronic medical record system. The association between UF and HRQoL was evaluated by ordered regression.

**Results:**

Seven-hundred-sixty-seven pregnant women with a mean age (SD) of 32.7 (4.8) years completed 707 questionnaires. Overall, when comparing the UF with non-UF groups, we detected statistical differences in age, body mass index (BMI), gravidity and abortion times, partner’s smoking and alcoholic habits, advanced maternal age, and uterine scars (*p* <  0.05). Furthermore, pregnant women without UF scored significantly higher than those with UF on the EQ-5D value system (0.84 versus 0.79; *p* = 0.017). Moreover, pregnant women with UF suffered more health-related problems, especially with respect to self-care (odds ratio [OR] = 3.69, *p* <  0.01) and usual activity dimensions (OR = 2.11; p = 0.01).

**Conclusion:**

We found that UF has a negative impact on the HRQoL of pregnant women with respect to self-care and usual activity dimensions. Also, the EQ-5D score was a better index than the EQ-VAS score for HRQoL when evaluating of the QoL of our population of pregnant women.

## Introduction

### Background

Uterine fibroids (UFs), also known as uterine myomas, fibromyomas, or leiomyomatas, are the most common benign tumors in women and have clinical morbidity rates of 20 to 40% and prevalence rates of 3 to 12% during pregnancy. The common causes of UFs are variable factors, such as genetics, endocrine factors and lifestyle factors [1–3]. The clinical outcomes range from asymptomatic to presentation of pain, menorrhagia, and obstetric complications such as infertility, miscarriage, and/or scarred uterus [3, 4]. However, these symptoms can affect the quality of life for pregnant women [5].

In a recent study, pregnant women were shown to suffer from an increase in the risk of depression [6], and depressive symptoms correlate with impairment of HRQoL [7]. According to prior studies, pain was shown to be the most common symptom of UF during pregnancy, and the risk of depression could increase in this situation [3, 8]. Some past studies used the EuroQoL Group’s five-dimension questionnaire (EQ-5D) to measure and assess the relationship between pain, depressive symptoms, and quality of life (QoL) [7, 9, 10]. Considering that most pregnant women do not have fatal diseases, it was suitable to use a generalized questionnaire to assess their HRQoL values. Therefore, as one of the most common instruments for measuring the overall body health state, the EQ-5D is a powerful and popular tool, especially for assessing pain and anxiety/depression symptoms [11, 12]. The symptoms and clinical outcomes caused by UFs might correlate with HRQoL; therefore, clinicians can use HRQoL as an index to evaluate the effectiveness of treatment [13, 14].

In contrast to traditional clinical outcomes, HRQoL during pregnancies with UFs can be used as an outcome indicator in health policy research, facilitating the improvement of clinical UF management. Utility data are key factors for cost-utility analysis and quality-adjusted life-year analysis in healthcare-related economics but have not been addressed in the literature; therefore, this study could provide a utility-based case value in pregnancies with UFs.

### Objectives

We aimed to evaluate several parameters: (1) determine the HRQoL in pregnancies with UFs; (2) provide a utility-based case value in pregnancies with UF; and (3) understand whether HRQoL is associated with the clinical outcomes of pregnant women with UFs.

## Method

### Study design

A cross-sectional study was performed as a part of a longitudinal project that studied pregnant women who received prenatal care during different gestational ages at one of the largest regional university hospitals in south China (The First Affiliated Hospital of the Sun Yat-sen University) from May 2017 to February 2018. Ethical approval was granted by the Institutional Review Board of The First Affiliated Hospital of Sun Yat-sen University (ICE-2017-296). All procedures were conducted in accordance with the Declaration of Helsinki. All participants signed the informed consent documents before participation in this study.

### Study population

All participants came from The First Affiliated Hospital of the Sun Yat-sen University. Eligible participants were included if they were pregnant. Only the first record for each participant was included in this study. Participants were excluded when they had missing personal information and/or clinical data. Furthermore, if any participants completes more than one EQ5D questionnaire, all additional records were excluded, except for the first one.

### Measurement

Patient-evaluated HRQoL is an important index in the assessment of a patient’s health and functional states [15]. The EuroQoL Group’s five-dimension questionnaire (EQ-5D) with EuroQoL Group’s visual analog scale questionnaire (EQ-VAS) is a common questionnaire for measuring the quality of life, making cost-efficiency calculations, and evaluating economic issues in the public health field. The EuroQoL Group’s five-dimension five-level questionnaire (EQ-5D-5 L) is a more reliable and sensitive instrument for measuring HRQoL than the EQ-5D-3 L [16]. The EQ-5D-5 L instrument contains a descriptive system for assessing a participant’s health state over five dimensions based on five levels in each dimension and utilizes a self-determined visual analog scale (VAS). These two parts were used throughout this study. The Chinese version of the EQ-5D-5 L has been shown to be valid and effective and is commonly used to measure HRQoL [17, 18]. EQ-VAS can provide a self-reported global measure of overall health and broader dimensions of assessment than EQ-5D although more than half the participants do not accurately evaluate themselves when using these types of scoring systems [19, 20]. This study might help to identify which of these two independent tools is more suitable for assessing pregnant women with UFs and provide detailed HRQoL data for future studies.

Participants were administered the EQ-5D questionnaire the first time that they visited the hospital for prenatal care. The EQ-5D assessed five dimensions (mobility, self-care, usual activity, pain/discomfort, and anxiety/depression) and it was based on five problem levels: (1) none; (2) slight; (3) moderate; (4) severe; and (5) extreme/unable. As examples, the self-care dimension asks about the degree of problems experienced when “washing and dressing by yourself”, and the usual activity dimension asks about the degree of problems in “work, study, housework, family, or leisure activities in daily life”. The five levels of response were represented by integer values (such as 1–5 with values of 2–5 indicating health-related problems) [16, 17, 19]. Each response pattern was calculated into a single EQ-5D index value (such as 11,221) through the EQ-5D-5 L Crosswalk Index Value Calculator to produce a final QoL value. The value ranged from − 0.224 to 1 with 1 indicating the best health state of people, whereas 0 represents death. Most patients are in the range from 0 to 1; however, it is still possible to achieve scores < 0, and these negative values correspond with overall health states (both physical and mental) that are considered worse than death [18]. We then measured each dimension and compared responses between pregnant women with and without UF. The EQ-VAS was a self-assessment of health state across five dimensions based on five levels of response. It presented as a vertical line with demarcations from 100 (best imaginable health state) to 0 (worst imaginable health state) [21]. Respondents were asked to draw a line from the bottom line 0 to the score line based on their opinion of their health states and fill the score in the blank beside.

### Variables

Basic independent covariates of the study population in the models included age, body mass index (BMI), living location, gravidity, parity, abortion, gestational trimester (first, second, or third), and advanced maternal age (indicated the age of pregnant women > 35 years old) [22]. A meta-analysis showed no significant impact of smoking on risk of UFs [23]. However, we wanted to detect whether the smoking state of pregnant women could affect the HRQoL in those pregnant women with UF. Furthermore, the partners’ lifestyle habits, such as smoking status and alcohol consumption, were also included in this study. Also, multipara, uterine scar, hepatitis B history, heart disease, and surgical history were included as part of the index for pre-pregnancy conditions. Throughout the study, participants were categorized by gestational trimester in which the first trimester was taken when the pregnant women were at the gestational age of < 13 weeks, the second was the gestational age of 13 to 28 weeks, and the third was a gestational age > 28 weeks. In generalized situations, we use transvaginal ultrasonography to detect and diagnose uterine fibroids. However, on rare occasions, such as suspected carcinoma (indicated by elevated cancer biomarker levels), a pathological examination might need to be performed to distinguish the uterine fibroids from uterine carcinoma under the current guideline in our hospital. In this situation, the risk of miscarriage due to the procedure needs to be balanced [24]. In this study, we did not have any cases that needed to undergo pathological examinations.

### Bias

The EQ-5D questionnaire was a subjective measurement of pregnant women’s HRQoL, and self-reported bias may be the main bias in this study. Based on the population, this study also minimized selection bias but had non-response, volunteer, and ascertainment biases.

### Statistical methods

Data analysis was performed using the STATA/SE 14.0 for Windows. Normally distributed continuous variables were described using the means + standard deviations (SDs), and ranges. Non-normal variables were presented as the median, and categorical variables were described using counts and percentages. The dependent variables were the EQ-5D score utility and EQ5D-VAS. EQ-5D scores were in a skewed distribution; therefore, we used a non-parametric approach to analyze the data.

Participants’ demographic data were reported (age, advanced maternal age, BMI, local, gravity, party, abortion, smoking, partner smoking status and alcoholic consumption, multipara, surgery history, hepatitis B, and heart disease). The clinical outcomes were retrieved from the hospital electronic medical system after delivery. Since the EQ5D values present a skewed distribution, we divided these values into two groups based for statistical analysis on the median EQ5D values: (1) above the median and (2) below the median [25]. Analysis of variance and t- and the chi-squared tests were used to compare continuous and qualitative variables among the three different trimesters. Health quality, as measured by the EQ5D-VAS scores or EQ5D values, and multiple linear regressions was used. Potential confounders were adjusted. An ordered logistic regression with odds ratios (ORs) and 95% confidence intervals (CIs) is an appropriate method to use when examining the effects of independent risk factors on various dimensions in the EQ5D index when complementary dimensions are taken into account [26, 27]. ORs, 95% CIs, and *p*-values were obtained using an ordered logistic regression analysis. All tests were two-sided, and a p-value of 0.05 was considered as statistically significant.

## Results

### Participants

**Fig. 1:**
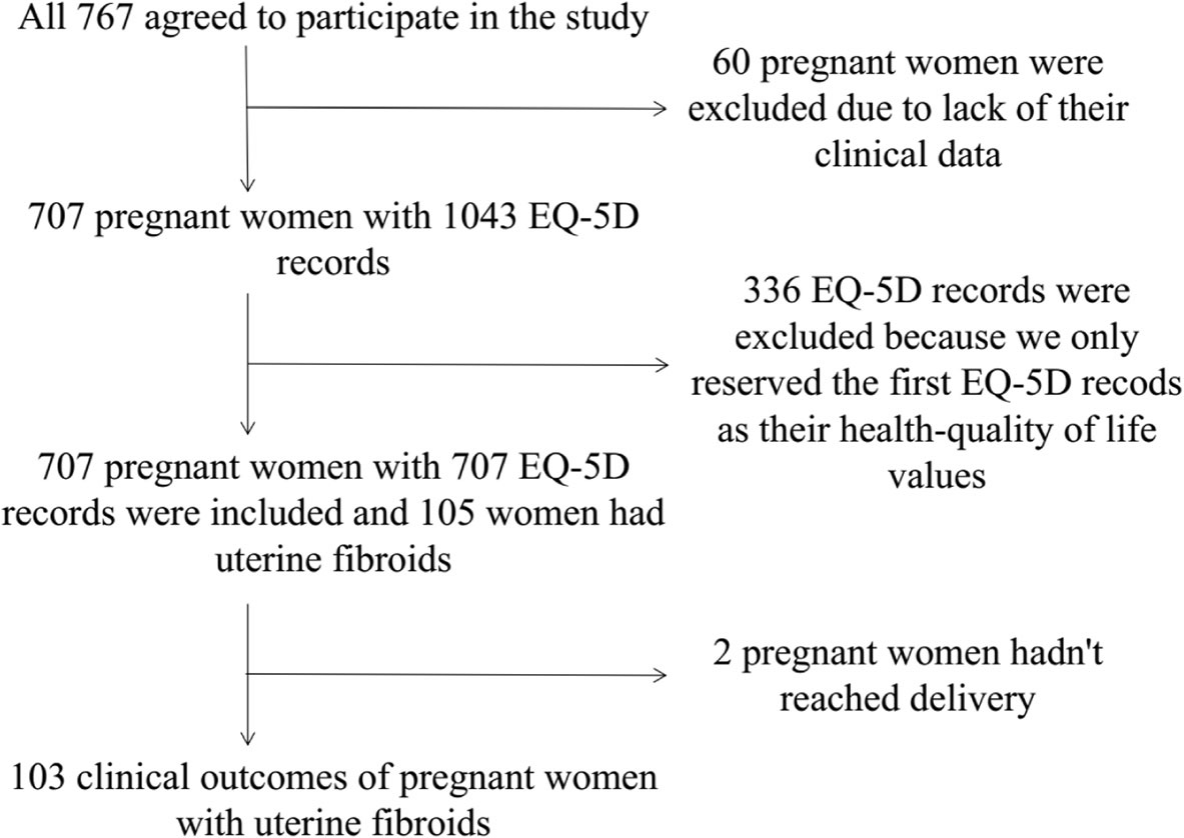
Selection of the study population

In total, all 767 pregnant women agreed to participate in this study, but of these 60 were excluded due to missing clinical data or personal information. We only reserved the first record as their HRQoL. Therefore, 707 of the included pregnant women provided 707 EQ-5D-5 L valid questionnaires for the analysis. Using electronic medical records, we identified 105 pregnant women with UFs. Of these, 103 were included in the study because two women did not deliver within the period under analysis (Fig. 1).

### Descriptive data

#### General characteristics of patients

Of 707 pregnant women, the ages ranged from 21.3 to 47.4 years, and the mean age was 32.7 ± 4.8 years. Of those, in UF group, 105 women (14.9%) were included and were 35.6 ± 4.8 years on average, while the non-UF group of pregnant women’ s mean age was 32.3 (4.5) years (Table 1). The mean BMI across those with UFs was 26.4 + 4.0 and was significantly lower (23.5 + 3.9) among those without UFs.

**Table 1:** Demographic characteristics

	UF (*n* = 105)	Non-UF (*n* = 602)	All patients (*n* = 707)	*p-value*
Age (SD)	35.6(4.8)	32.3(4.5)	32.7(4.8)	**< 0.01**
Advanced maternal age	53(50.5%)	161(26.7%)	214(30.3%)	**< 0.01**
BMI (SD)	26.4(4.0)	23.5(3.9)	23.9(4.1)	**< 0.01**
Living location (Guangzhou)	96(91.4%)	547(90.9%)	643(90.9%)	0.85
Gravidity (SD)	2.4(1.2)	2.1(1.1)	2.2(1.1)	**0.03**
Parity (SD)	0.6 (0.5)	0.5(0.5)	0.5(0.5)	0.57
Abortion (SD)	0.8(0.9)	0.6(0.8)	0.6(0.9)	**0.01**
Smoking	0(0.0%)	0(0.0%)	0(0.0%)	–
Partner Smoking	24(22.9%)	85(14.1%)	109(15.4%)	**0.02**
Partner Alcoholics	14(13.3%)	43(7.1%)	57(8.1%)	**0.03**
Multipara	5(4.8%)	101(16.8%)	106(15.0%)	**< 0.01**
Surgical history	26(24.8%)	149(24.8%)	175(24.8%)	1.00
Uterine scar	33(31.4%)	131(21.8%)	164(23.2%)	**0.03**
Hepatitis B	1(1.0%)	14(2.3%)	15(2.1%)	0.37
Heart disease	1(1.0%)	5(0.8%)	6(0.9%)	0.90

*UF* Uterine fibroids, *BMI* Body mass index. Bold represented *p* value < 0.05. Data with SD indicates “mean” value. Data without SD indicates “number”

**Table 2:** Clinical outcomes of pregnant women with uterine fibroids

	Below median EQ5D score (*n* = 52)	Above median EQ5D score (*n* = 51)	All UF patients (*n* = 103)	*p*-value
Cesarean Section	39(75.0)	31(60.8)	70(68.0)	0.91
Preterm labor	5(9.6)	9(17.6)	14(13.6)	0.67
Precipitate labor	0(0.0)	3(5.9)	3(2.9)	0.11
Placenta adherence	8(15.4)	7(13.7)	15(14.6)	0.34
Nuchal cord around neck	10(19.2)	15(29.4)	25(24.3)	0.19
PROM	11(21.2)	15(29.4)	26(25.2)	0.28
Postpartum hemorrhage	1(1.9)	2(3.9)	3(2.9)	0.44
Amniotic fluid turbidity	6(11.5)	6(11.8)	12(11.7)	0.31
Perineal laceration	7(13.5)	10(19.6)	17(16.5)	0.83
Hypertensive disorders	14(26.9)	9(17.7)	23(22.3)	0.90
Gestation age at birth	37.8(1.4)	38.1(1.9)	37.9(1.7)	0.73
Apgar score - 1 min	9.85(0.5)	9.75(0.63)	9.8(0.57)	0.13
Fetal distress	5(9.6)	12(23.5)	17(16.5)	0.07

*PROM* Premature rupture of membrane

Of the 707 eligible pregnant women, most (90.9%) lived locally (living in Guangzhou). The mean values for gravidity, parity, and abortion times across the entire population were 2.2 ± 1.1, 0.5 ± 0.5, and 0.6 ± 0.9, respectively. All 707 pregnant women denied smoking during pregnancy. Some women’s partners (15.4%) were smokers, and 8.1% of the partners consumed alcohol. Fifteen percent of the sample population was multipara, 24.8% had surgical histories, 30.3% were of advanced maternal age, 23.2% had scars on the uterus, 2.1% had hepatitis B, and 0.9% had chronic heart disease. Table 1 shows these risk factors for the sample population. Significant differences were found in age between those with and without UFs (*p* 0.01), BMI (*p* < 0.01), gravidity (*p* = 0.03), abortion (*p* = 0.01), partner smoking (*p* = 0.02), partner alcoholism (*p* = 0.03), scarred uterus (*p* = 0.03), multipara (*p* < 0.01), and advanced maternal age (*p* < 0.01).

### Outcome data

#### The characteristics of clinical outcomes in pregnant women with UFs

Two of the study participants did not deliver at the time point of analysis. Thus, these results only included clinical outcomes of 98.15% (103 out of 105) pregnant women with UFs. Among those pregnant women enrolled in the study (except for the two that had not delivered), about 44.7% belonged to the below median EQ-5D score group while 55.3% came from the above median EQ-5D score group (Table 2). The mean + SD gestational ages for the below median and above median EQ-5D score groups were 37.8 + 1.7 and 38.0 + 1.6 weeks, respectively. For pregnancy complications, no statistical difference was found between two groups.

### Main results

#### The EQ-5D and EQ-VAS values assessed using the EQ-5D-5 L

**Fig. 2:**
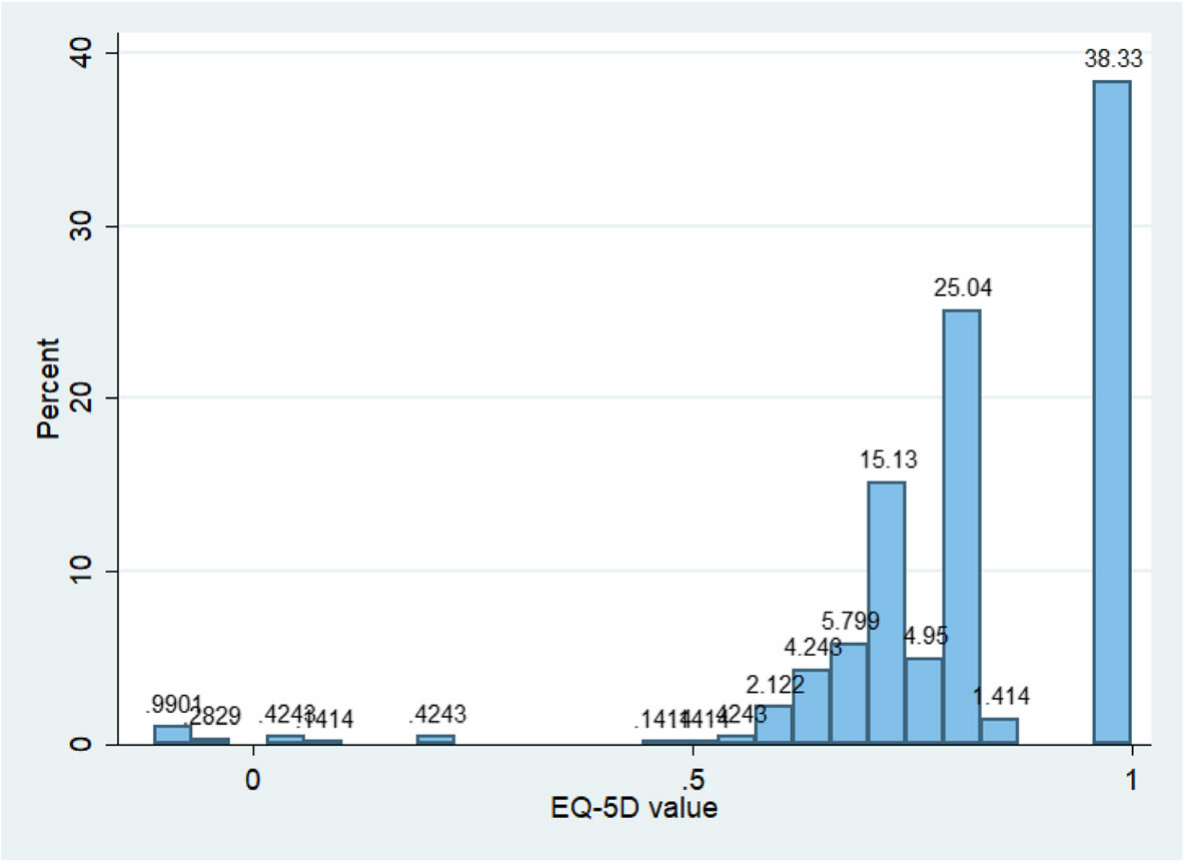
Distribution of EQ-5D values of all pregnant women

**Fig. 3:**
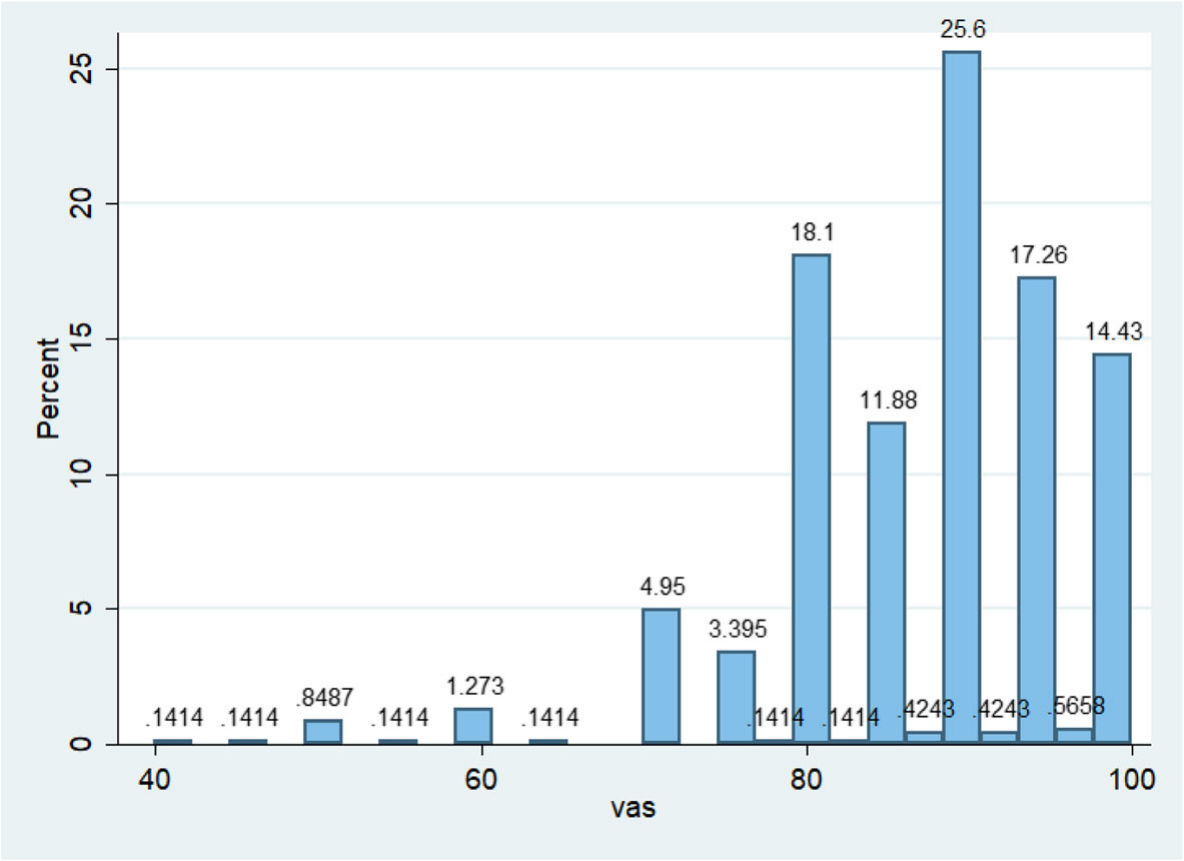
Distribution of EQ5D-VAS values of all pregnant women

The EQ-5D and EQ-VAS value distributions are shown (Figs. 2 and 3). Of the total 707 EQ-5D and EQ-VAS records (707 pregnant women), the mean (SD) EQ-5D indices for those with and without UFs were 0.79 ± 0.21 and 0.84 ± 0.18, respectively (*p* = 0.017) and the mean of EQ-VAS with and without UFs were 88.0 ± 8.6 and 87.3 ± 9.9, respectively (*p* = 0.480) (Table 3). Besides, the groups also showed differences in age and BMI (both *p* <  0.01) (Table 1). Therefore, age and BMI were adjusted in the analysis. After adjustment, EQ-5D scores were significantly different between women with and without UFs (*p* = 0.007), while the EQ-VAS score showed no statistically significant difference between the two groups (*p* = 0.486).

**Table 3:** EQ-5D Index and EQ-VAS scores with and without UF

	EQ5D Index (*n* = 707)		
	Unadjusted	Age adjust	Age & BMI adjusted
UF group	0.79(0.21)	0.79(0.00)	0.79(0.00)
Non-UF group	0.84(0.18)	0.84(0.00)	0.84(0.00)
*p-value*	0.017	0.002	0.007
	EQ5D-VAS (*n* = 707)		
	Unadjusted	Age-adjusted	Age & BMI adjusted
UF group	88.0(8.6)	88.0(0.01)	88.0(0.01)
Non-UF group	87.3(9.9)	87.2(0.00)	87.3(0.00)
*p-value*	0.480	0.522	0.486

*UF* Uterine fibroids, *BMI* Body mass index

**Fig. 4:**
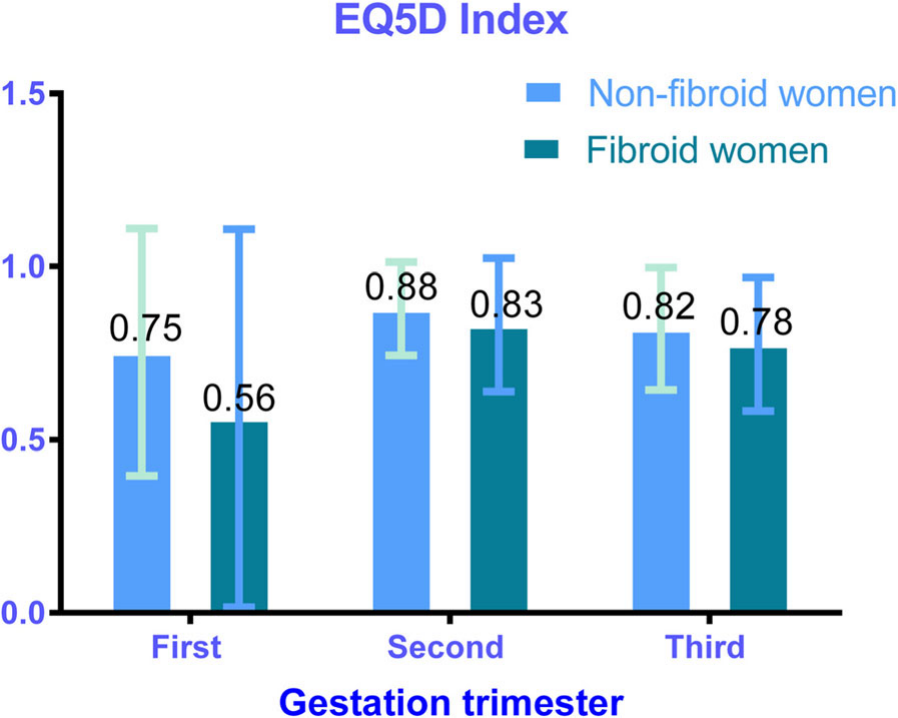
EQ5D index and its 95% confidence interval (CI) on gestational trimesters. a.The first-trimester group contains 30 pregnant women, 27 pregnant women without UFs, and 3 pregnant women with UFs. b.The second-trimester group contains 263 pregnant women, 220 pregnant women without UFs, and 43 pregnant women with UFs. c.The third-trimester group contains 414 pregnant women, 355 pregnant women without UFs, and 59 pregnant women with UFs. d.UFs indicates uterine fibroids

**Fig. 5:**
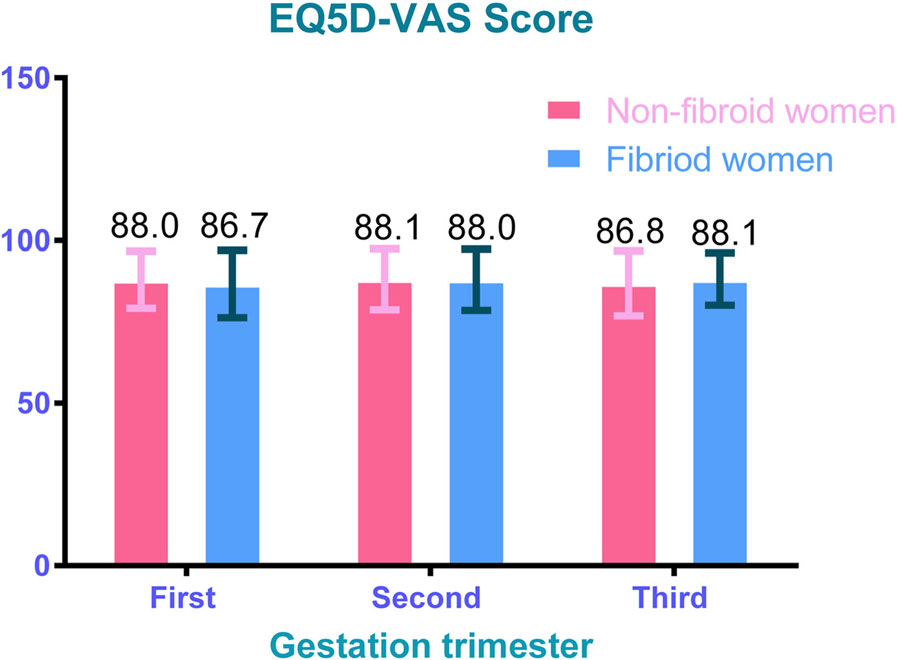
EQ5D-VAS scores and its 95% confidence interval (CI) on gestational trimesters. a. The first-trimester group contains 30 pregnant women, 27 pregnant women without UFs, and 3 pregnant women with UFs. b The second-trimester group contains 263 pregnant women, 220 pregnant women without UFs, and 43 pregnant women with UFs. c The third-trimester group contains 414 pregnant women, 355 pregnant women without UFs, and 59 pregnant women with UFs. d UFs indicates uterine fibroids

The EQ-5D and EQ5D-VAS scores varied across the different gestational trimesters (Figs. 4 and 5). Pregnant women with UFs scored lower indices on the EQ-5D compared to those without UFs, regardless of the trimester. Among those without UFs, the mean EQ5D indices were 0.75, 0.88, and 0.82 in the first, second, and third trimesters, respectively. In the first, second, and third trimesters, mean EQ5D indices were 0.56, 0.83, and 0.78, respectively, among those pregnant women with UF. The mean EQ5D-VAS scores were lower among those with UFs compared to those without, except in the third trimester. Also, women from both groups (non-UF versus UF) presented the greatest EQ-5D indices (0.88 versus 0.83) and EQ5D-VAS scores (88.1 versus 88.0) in the second trimester.

#### Uterine fibroids and other factors contributing to health quality

The 707 EQ-5D records were classified based on the presence of UFs in the participant. Table 4 shows that 23.8% of the records for those with UFs also indicated problems with mobility, 20.0% with self-care, 29.5% with usual activity, 52.4% with pain/discomfort, and 35.2% with anxiety/depression. Additionally, 18.9% of the pregnant women without UFs suffered mobility problems, 7.3% had self-care problems, 16.0% had problems with their usual activities, 45.7% had pain/discomfort problems, and 30.1% had anxiety/depression problems. The results indicate that pain and discomfort during pregnancy were major problems for pregnant women, while problems with self-care were of the least concern. Besides, there were about 13.5% more health-related problems with respect to the usual activity dimension in the UFs group than that in non-UFs group, which was a noticeable difference between pregnant women with and without UFs for the five dimensions. For the overall status, there was a greater proportion of those with UFs who experienced health-related problems (regardless of the dimensions) when compared to those without UFs.

**Table 4:** The frequencies of pregnant women that report levels 1 to 5 for the various dimension

EQ-5D Dimension		Uterine fibroid		Total (%)
		UF (%)	Non-UF (%)	
Mobility	Level 1	80(76.2)	488(81.1)	568(80.3)
	Level 2	20(19.1)	91(15.1)	111(15.7)
	Level 3	1(1.0)	9(1.5)	10(1.4)
	Level 4	0(0.0)	3(0.5)	3(0.4)
	Level 5	4(3.8)	11(1.8)	15(1.4)
Self-care	Level 1	84(80.0)	558(92.7)	642(90.8)
	Level 2	17(16.2)	29(4.8)	46(6.5)
	Level 3	1(1.0)	2(0.3)	3(0.4)
	Level 4	0(0.0)	1(0.2)	1(0.1)
	Level 5	3(2.9)	12(2.0)	15(2.1)
Usual Activity	Level 1	74(70.5)	505(84.0)	579(81.9)
	Level 2	26(24.8)	75(12.5)	101(14.3)
	Level 3	2(1.9)	11(1.8)	13(1.8)
	Level 4	0(0.0)	3(0.5)	3(0.4)
	Level 5	3(2.9)	8(1.3)	11(1.6)
Pain/Discomfort	Level 1	50(47.6)	327(54.3)	377(53.3)
	Level 2	49(46.7)	246(40.9)	295(41.7)
	Level 3	3(2.9)	15(2.5)	18(2.6)
	Level 4	0(0.0)	6(1.0)	6(0.9)
	Level 5	3(2.9)	8(1.3)	11(1.6)
Anxiety/ Depression	Level 1	68(64.8)	421(69.9)	489(69.2)
	Level 2	34(32.4)	157(26.1)	191(27.0)
	Level 3	0(0.0)	11(1.8)	11(1.6)
	Level 4	1(1.0)	4(0.7)	5(0.7)
	Level 5	2(1.9)	9(1.5)	11(1.6)

An ordered logistic regression analysis was used for each dimension in the EQ-5D (Table 5). Women in their second or third trimesters reported more problems with mobility (OR = 1.77; *p* < 0.01) and pain/discomfort (OR = 1.46; *p* < 0.01) than those in their first trimester. UFs were related to self-care problems (OR = 3.69; *p* < 0.01) and usual activity problems (OR = 2.11; *p* < 0.01). Pregnant women who were not located locally suffered from more severe pain/discomfort problems during the first trimester (OR = 2.16; *p* < 0.01). More gravidity was associated with problems regarding usual activity (OR = 1.33; *p* = 0.01), and less parity was associated with problems with usual activity (OR = 0.56; *p* = 0.03) and anxiety/depression (OR = 0.60; *p* = 0.02).

**Table 5:** Ordered logistic regression analysis for each dimension in the EQ5D

		Odds ratio	95% CI	*p-value*
Mobility	Age	0.98	0.93, 1.02	0.35
	BMI	1.03	0.98, 1.08	0.26
	Local	0.83	0.48, 1.43	0.50
	Gestation trimester	1.77	1.21, 2.60	**< 0.01**
	Partner smoking	1.10	0.64, 1.87	0.74
	Partner Alcoholics	1.21	0.59,2.51	0.60
	Gravidity	1.17	0.95, 1.45	0.15
	Parity	0.97	0.60, 1.57	0.90
	UF	1.32	0.77, 2.25	0.31
Self-care	Age	0.96	0.90, 1.02	0.17
	BMI	1.00	0.94, 1.08	0.94
	Local	1.15	0.48, 2.76	0.16
	Gestation trimester	1.58	0.95, 2.65	0.08
	Partner smoking	0.74	0.38, 1.42	0.36
	Partner alcoholics	1.79	0.59, 5.37	0.30
	Gravidity	0.91	0.64, 1.28	0.59
	Parity	0.72	0.35, 1.49	0.38
	UF	3.69	1.94, 7.03	**< 0.01**
Usual activity	Age	0.98	0.93, 1.03	0.47
	BMI	1.03	0.97, 1.08	0.34
	Local	0.74	0.43, 1.29	0.29
	Gestation trimester	1.38	0.94, 2.00	0.10
	Partner smoking	1.46	0.80, 2.66	0.22
	Partner alcoholics	1.16	0.54, 2.49	0.71
	Gravidity	1.33	1.07, 1.66	**0.01**
	Parity	0.56	0.33, 0.94	**0.03**
	UF	2.11	1.25, 3.55	**< 0.01**
Pain/Discomfort	Age	0.96	0.93, 1.00	0.06
	BMI	1.00	0.96, 1.04	0.96
	Local	2.16	1.26, 3.71	**< 0.01**
	Gestation trimester	1.46	1.11 1.93	**< 0.01**
	Partner smoking	1.26	0.82, 1.93	0.29
	Partner alcoholics	0.71	0.41, 1.26	0.25
	Gravidity	1.07	0.90, 1.29	0.45
	Parity	0.75	0.51, 1.10	0.14
	UF	1.47	0.94, 2.28	0.09
Anxiety/depression	Age	1.00	0.96, 1.04	0.93
	BMI	1.01	0.97, 1.06	0.55
	Local	1.07	0.65, 1.78	0.78
	Gestation trimester	0.90	0.68, 1.21	0.49
	Partner smoking	0.85	0.56, 1.31	0.47
	Partner alcoholics	0.97	0.53, 1.76	0.91
	Gravidity	1.04	0.86, 1.26	0.68
	Parity	0.60	0.39, 0.91	**0.02**
	UF	1.18	0.74, 1.89	0.75

Bold represented *p-value* < 0.05

## Discussion

### Key results

The major finding of the present study indicated that uterine fibroids could significantly affect HRQoL of pregnant women in two dimensions (self-care and usual activities) when compared to women without UF. Pregnant women with UFs had a lower EQ-5D index than those without UFs (0.80 versus 0.84) while their average EQ5D-VAS scores were 88.0 versus 87.3. This EQ-VAS result was similar to that found in other research in China in which evaluated health status in a similarly-aged cohort was evaluated [28, 29]. Less than 15% of pregnant women rated their health status as 100 (best possible health state) based on the EQ5D-VAS. However, the EQ-VAS scores did not show statistically significant differences between pregnant women with and without UFs, and the different standards of health self-evaluation from each participant could cause bias in the EQ5D-VAS results; thus, the EQ5D index might be a more suitable index than the EQ-VAS for evaluating HRQoL in pregnant women with UFs. Furthermore, pregnant women showed the greatest EQ-5D indices and EQ5D-VAS scores during their second gestational trimester regardless of UF or non-UF group (Figs. 4 and 5). Figure 2 shows that the EQ-5D value was the lowest in the first gestational trimester regardless of UF status. However, this finding was not in agreement with results of some other published studies [30, 31]. This lack of agreement might be caused by the policy of performing a full systemic prenatal examination and consultation in the second and third trimesters in China in addition to health-care appointments every 2 or 4 weeks, which might improve pregnant women’s the HRQoL. Those with UFs had more health-related problems across a range of five dimensions (mobility, self-care, usual activity, pain/discomfort, and anxiety/depression) compared to those without (Table 4). About half of the pregnant women (46.7%) suffered health problems associated with pain and discomfort; this was the greatest proportion of the five dimensions. Thus, it is necessary for the public healthcare system to focus on relieving this pain and discomfort when designing policies.

Besides, the EQ5D indices with respect to mobility and pain/discomfort dimensions fas reported by women without UFs were greater than those with UFs regardless of the trimester, which might be explained by lower levels of physical activities during the second and third trimesters [32]. Increased gravidity significantly correlated with the rising odds of usual activity problems (Table 5) because pregnant women who have increased gravidity in China mostly had their second child at advanced maternal ages because of the recent start of the Chinese two-child policy and long period of China’s one-child policy. Under these conditions, pregnant women with advanced maternal ages were more likely to receive more medical care during pregnancy, which might be explain the increase in usual activity problems. In addition, decreased parity (mostly nulliparity) could contribute to the increase in usual activity problems. A previous study had clearly recognized a decrease in parity as a risk factor for the incidence of UFs [24], possibly because the production of estrogen and progesterone declines in parity and has considerable effects on fibroids’ growth [33].

These findings offer additional and detailed evidence for the negative influence of UFs on HRQoL, which is in agreement with other cross-sectional studies. One web-based cross-sectional study investigated the HRQoL across 4848 women aged 18 to 49 years using the Uterine Fibroid Symptom-Quality of Life Questionnaire (UFS-QoL) and demonstrated a significant reduction in HRQoL in women with UFs [34]. An online cross-sectional study also found that the HRQoL might decrease with UFs and might be significantly impacted by UF-related symptoms [35]. Our study shows that UFs in pregnant women might affect the HRQoL scores in the self-care and usual activity dimensions (Table 5).

Comparative assessments of pregnant women with and without UFs based on gestational index and chronic conditions indicate that some of these independent factors contributed considerably to the incidence of UFs, while other conditions were in an inverse relation with UFs. Ages and BMIs are higher among those with UFs than those without, which can be explained by a greater age indicating more gravidity, abortion, and opportunities for pregnancy during advanced maternal age (Table 1) [1, 36]. The incidence of UFs (*p* < 0.05) will cause higher gravidity and abortion rates according to our data (Table 1), and this finding could be explained by the fact that UFs can significantly lead to more infertility [3]; therefore, gravidity and abortion rates will increase correspondingly. We were surprised about the self-report smoking status of the pregnant women as none of them smoked. This finding might be due to the family planning policy (one-child policy in the past and two-child policy recently) in China, which might increase mothers’ concerns about their baby’s health status although self-report bias is another possible explanation. Also, it seemed that there was no statistical significantly impact between partner smoker/alcohol consumption and pregnant women’s HRQoL state in the different dimensions (Table 5).

One prior study demonstrated that the rate of Cesarean section has increased from 28.8% in 2008 to 34.9% in 2014 in China [37], and this rate was shown to be related to family income, education, health insurance, Chinese health policy, and cultural background, among other factors [38]. In pregnant women with UFs, Cesarean section rates reached 67.0%, which was much higher than the rate in the normal population worldwide. Cesarean section may be the most suitable management for pregnant women with other pre-pregnancy conditions [39]. These findings are consistent with prior published data [35, 40–43].

Although there was no significant correlation between low HRQoL and poor clinical outcomes of pregnant women, further studies need to be done to verify these results. QoL is becoming an increasingly important indicator of the effectiveness of the medical intervention, and we should pay greater attention to QoL during pregnancy in our future practice.

### Limitations

There were some limitations to this study. This was a cross-sectional study, and the data was obtained from an EQ-5D questionnaire in which there was relatively a subjective measurement of pregnant women’s HRQoL. Thus, self-report bias may be the main bias in this study. This study design also presents limitation with respect to both non-response and volunteer biases. Furthermore, we do not make comparisons against different instrument other than the EQ-5D. There are some studies that have used the UFS-QoL to assess the HRQoL in pregnant women [34, 35]. This research could provide HRQoL data for pregnant women who were evaluated using the EQ-5D-5 L, and this information could be useful in cost-utility analyses in the healthcare-related economic area. Additionally, the HRQoL is an important indicator of a patient’s overall state and plays an increasingly important role in evaluation in the clinic although there is no significant difference with respect to clinical outcomes between women with and without UFs in the short-term. Nevertheless, we believe that better quality life-related studies should be performed in order to further investigate the role of QoL in the clinic and monitor long-term effects on QoL. Future studies will need to use a cohort to observe the changes in HRQoL in pregnant women.

### Interpretation

To our knowledge, this is the first clinical study to use the EQ-5D-5 L to focus on HRQoL during pregnancy in China and detect independent factors that could impact HRQoL in pregnant women with UFs. Also, this study shows that EQ-5D index may be a better index than EQ-VAS for pregnant women with uterine fibroids and possibly for other medical comorbidities or complications. Furthermore, the HRQoL data assessed by the EQ-5D-5 L could be used to perform cost-utility analyses in the future. The clinical outcomes of pregnant women with UFs could still offer insight for the clinical physician when considering the possibility of latent complications in pregnant women with UFs.

## Conclusion

In this study, we evaluated the influence of UFs on HRQoL in pregnant women and found that the EQ5D assessment instrument outperformed the EQ-VAS. Our findings demonstrated that UFs significantly affected HRQoL in pregnant women in terms of the self-care and usual activity dimensions. Independent factors, such as living locally, gravidity and parity times, and gestational trimester, could have a significant impact on the HRQoL. Finally, whether clinical outcomes may affect HRQoL scores need to be precisely analyzed and requires further research.

## Abbreviations

BMI: Body mass index
CIs: Confidence intervals
EQ-5D: The EuroQoL Group’s five-dimension questionnaire
EQ-5D-3 L: The EuroQoL Group’s five-dimension three-level questionnaire
EQ-5D-5 L: The EuroQoL Group’s five-dimension five-level questionnaire
EQ-VAS: EuroQoL Group’s visual analog scale questionnaire
HRQoL: Health-related quality of life
ORs: Odds ratios
PROM: Premature rupture of membrane
QoL: Quality of lfe
UFs: Uterine fibroids
UFS-QoL: Uterine Fibroid Symptom-Quality of Life
VAS: Visual analog scale
